# Is Low-Frequency Electrical Stimulation a Tool for Recovery after a Water Rescue? A Cross-Over Study with Lifeguards

**DOI:** 10.3390/ijerph17165854

**Published:** 2020-08-12

**Authors:** Roberto Barcala-Furelos, Alicia González-Represas, Ezequiel Rey, Alicia Martínez-Rodríguez, Anton Kalén, Olga Marques, Luís Rama

**Affiliations:** 1 REMOSS Research Group, Faculty of Education and Sport Sciences, University of Vigo, 36005 Pontevedra, Spain; roberto.barcala@uvigo.es (R.B.-F.); anton.kalen@gmail.com (A.K.); 2 CLINURSID Network Research, Department of Psychiatry, Radiology and Public Health, University of Santiago de Compostela, 15782 Santiago de Compostela, Spain; 3 Department of Functional Biology and Health Sciences, Faculty of Physiotherapy, University of Vigo, 36005 Vigo, Spain; alicia@uvigo.es; 4 Department of Physiotherapy, Medicine and Biomedical Sciences, Universidade da Coruña, 15006 La Coruña, Spain; alicia.martinez@udc.es; 5 Faculty of Sports Sciences and Physical Education, University of Coimbra, 3040-156 Coimbra, Portugal; olgagaboleiro@gmail.com (O.M.); luisrama@fcdef.uc.pt (L.R.); 6 Research Unit for Sport and Physical Activity (CIDAF), 3040-156 Coimbra, Portugal

**Keywords:** transcutaneous electric nerve stimulation, task performance and analysis, tensiomyography, lactate, lifesaving

## Abstract

This study aimed to evaluate the degree to which transcutaneous electrical stimulation (ES) enhanced recovery following a simulated water rescue. Twenty-six lifeguards participated in this study. The rescue consisted of swimming 100 m with fins and rescue-tube: 50 m swim approach and 50 m tow-in a simulated victim. Blood lactate clearance, rated perceived effort (RPE), and muscle contractile properties were evaluated at baseline, after the water rescue, and after ES or passive-recovery control condition (PR) protocol. Tensiomiography, RPE, and blood lactate basal levels indicated equivalence between both groups. There was no change in tensiomiography from pre to post-recovery and no difference between recovery protocols. Overall-RPE, legs-RPE and arms-RPE after ES (mean ± SD; 2.7 ± 1.53, 2.65 ± 1.66, and 2.30 ± 1.84, respectively) were moderately lower than after PR (3.57 ± 2.4, 3.71 ± 2.43, and 3.29 ± 1.79, respectively) (*p* = 0.016, *p* = 0.010, *p* = 0.028, respectively). There was a significantly lower blood lactate level after recovery in ES than in PR (mean ± SD; 4.77 ± 1.86 mmol·L^−1^ vs. 6.27 ± 3.69 mmol·L^−1^; *p* = 0.045). Low-frequency ES immediately after a water rescue is an effective recovery strategy to clear out blood lactate concentration.

## 1. Introduction

Lifeguards require a high level of physical conditioning to respond to the physiological demands of a water rescue [1]. Drowning is a global health problem, which in 2017 caused 295,210 drowning deaths [2], a number that would be much higher without lifeguard prevention and rescues. Drowning is triggered, within seconds or minutes [3], so the intensity of lifeguards’ water rescues is very high, with lactate production of over 10 millimoles [4,5] and great physiological and muscular fatigue [6]. Considering a lifeguard can have more than one rescue per day [7], the physiological and metabolic disturbances induced by a water rescue can have detrimental effects on the performance of subsequent rescues. Thus, lifeguards need to recover effectively between events [6].

Several post-exercise strategies are available to lifeguards in order to accelerate the recovery process after a rescue [6]. However, despite their use in practice, the scientific evidence of their efficacy in lifesaving is limited. Electrical stimulation (ES) could be a potentially useful method for lifeguards to accelerate the recovery process and thereby their ability for a subsequent rescue. ES has been used previously in swimming for recovery after intense efforts [8]. This recovery process could even take place from a watchtower, while the lifeguard surveils the beach. It has been suggested that low-frequency ES could enhance recovery because of two main mechanisms: increasing blood flow due to the muscle pump effect, or reducing the muscle pain through central and peripheral mechanisms [9]. Motor-level, low-frequency ES at 5 Hz has shown to improve venous return in healthy people [10], and high amplitudes (strong not painful intensities) may be the key for acute pain relief [11]. In addition, ES can increase both peak venous velocity and venous volume flow up to over 600% [12] and improve microcirculation, so it may help reduce inflammation or edema [13] after intense exercise.

Another important expected effect of ES used for post-exercise recovery is the restoration of neuromuscular properties [9]. However, previous literature showed conflictive findings regarding muscular parameters restoration after ES application [9]. Thus, further research is needed to solve the ambiguity of the evidence on this topic. In this context, tensiomyography (TMG) is a very sensitive, simple, and noninvasive method for measuring muscle contractile properties that has been identified as a potential tool for assessing post-exercise stiffness and neuromuscular status [14,15]. This measurement is carried out under isometric conditions, in response to an electrical stimulus, and could provide important information on acute muscular responses to different exercise stimulus. Specifically, several investigations have highlighted the usefulness of TMG variables in detecting muscular changes following various kinds of exercise [16,17] and recovery methods [18,19,20,21]. In relation to recovery methods, published research has produced conflictive results regarding the effectiveness of different recovery strategies on TMG variables. Low-frequency vibration recovery method and active recovery failed to demonstrated efficacy on restore TMG parameters after a cycling fatiguing exercise [18] and a soccer training session [19], respectively. However, muscle contractile properties assessed with TMG cold-water immersion were recovered after different water-immersion recovery protocols in healthy men [20] and soccer players [21].

Thus, this study aimed to examine whether ES is an effective recovery tool to aid the recovery of beach lifeguards. According to the aforementioned considerations, the following hypotheses were tested: (1) ES would promote greater recovery effects in perceived fatigue compared with a passive-recovery control condition (PR); (2) ES would promote greater blood lactate clearance compared with PR; and (3) ES would not minimize neuromuscular fatigue compared with PR.

## 2. Materials and Methods

### 2.1. Experimental Design

**Figure 1:**
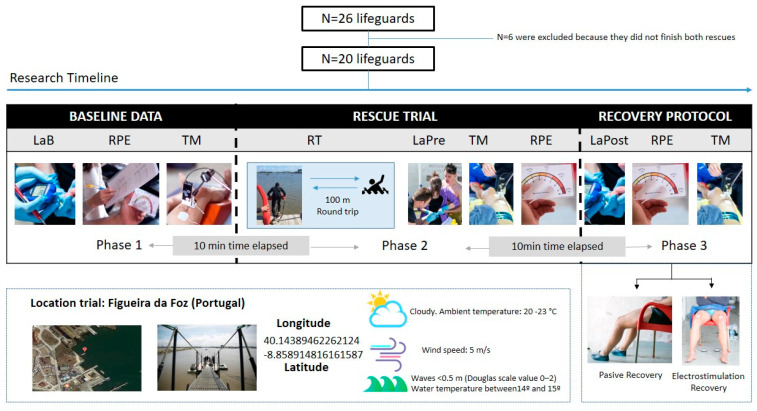
Trial flow chart. LaB = Lactate (Baseline), RPE = rate of perceived exertion, TM = tensiomyography, RT = rescue time, LaPre = Lactate Pre-recovery protocol, and LaPost = Lactate Post-recovery protocol.

A quasi-experimental cross-over study design was used to test the effectiveness of one recovery method, low-frequency ES, compared with seated resting, as a typical passive recovery (PR) strategy, on the blood lactate clearance, perceived effort, and muscle contractile properties after performing a water rescue (Figure 1). Participants were randomly allocated to either recovery protocol sequence. The assigned groups were determined by a chance process (a random number generator on a computer) and could not be predicted.

The research was approved by the Ethical Committee of the Faculty of Sport Sciences and Physical Education of University of Coimbra (Portugal), code CE/FCDEF-UC/00312018, in accordance with Helsinki declaration.

### 2.2. Participants

A convenience sample of 26 lifeguards (84.6% were men) from Figueira da Foz, Coimbra, Portugal participated in the experiment (age: 24.0 ± 4.9 years, height: 175.3 ± 7.1 cm, weight: 74.8 ± 10.0 kg, BMI: 24.0 ± 2.5). A total of 20 participants completed both recovery methods and were included in subsequent analyses. Six lifeguards did not complete the two rescues. Four of them were discarded for not being able to meet the protocol times. Two lifeguards left the study without performing the second rescue. All participants gave written informed consent prior to the study.

### 2.3. Procedures

The water rescue consisted of swimming 100 m with fins to the victim, gaining control of the conscious non-collaborative victim, and towing the victim 100 m back to shore using a common rescue tube (Size: 50″ × 6″ × 4″). This distance has been used in other investigations with lifeguards [5,22]. The participating victims (height: 160–190 cm, weight: 60–90 kg) were instructed to simulate a conscious person without collaboration. Lifeguards used their fins and wetsuit (as in a real rescue).

The rescues were all performed at Figueira da Foz port, Portugal (Latitude: 40.147954, Longitude: −8.859334) under similar conditions: calm sea without waves 0.5 m (Douglas scale value 0–2), wind speed b5 m/s, water temperature ranged between 14 °C and 15 °C, and ambient temperature between 20 °C and 23 °C. The weather was reported by the local forecast agency.

Rescue lactate level and rate of perceived exertion (RPE) were collected directly after the participants finished the water rescue. The participants then immediately removed their wetsuits whereby the rescue tensiomigrophy measures were taken. After rescue measures had been taken, the participants proceeded with the corresponding recovery protocol. The total time between the initiation of rescue measures and recovery measures was 25 min. 

For the resting recovery protocol, the participants remained seated for the full recovery period. The PR protocol simulates an immediate return of the lifeguard to a seated position in the watchtower, which very likely is the most common form of recovery for lifeguards today.

The participant’s skin was shaved 48 h before or was clipped of hairs when needed, and 70° alcohol was applied to the stimulation sites. A portable transcutaneous electrical nerve stimulation device (DUO TENS, Globus, Italy) and self-adhesive surface electrodes (Durastick Premium) were used. Two electrodes (5 × 9 cm) were used for each quadriceps. The positive electrode (anode) was placed proximally, just below the trochanter, with its midline aligned with the anterosuperior iliac spine (ASIS). The cathode was placed distally with the upper edge at the beginning of the distal 1/3 of the line from ASIS to the patella, with the midline of the electrode situated at the inner edge of the patella. The electrodes (5 × 5 cm) from the third channel were applied on both solei (cathode on the dominant leg), 5 cm distal from where the two heads of the gastrocnemius join the Achilles tendon. A 20-min charge-balanced biphasic square wave at 5 Hz with a phase duration of 0.25 milliseconds was employed. The current amplitude was set beyond the motor threshold at the maximum comfortable level (without pain) [10]. Every 5 min, the participant’s sensation was reassessed, and if it had decreased, then the amplitude was increased until the participant reported the same feeling last 5 min. The mean (standard deviation) amplitude level at the five last minutes of ES was 24.07 (7.48) mA and 31.14 (9.67) mA, for quadriceps and soleus muscles, respectively.

Tensiomyography (TMG-S1 model) was used to evaluate muscle responses and the effects of recovery methods used in this study. Measurements were made on the *rectus femoris* muscle of the dominant leg under static and relaxed conditions at baseline (a measurement prior to the beginning of the tests), after the water rescue and after recovery. With the participant in the supine position, the knee joint was fixed at a 120° angle (180° corresponding to full extension of the knee). The measured limb was positioned on a triangular wedge foam cushion to keep a fixed knee angle. A Trans-Tek^®^ DC-DC digital displacement transducer (GK 40, Panoptik d.o.o., Ljubljana, Slovenia), which incorporates a spring of 0.17 N·m^−1^, was set perpendicular to the muscle belly. The measuring point for each muscle was anatomically established as the point of maximal muscle belly displacement detected by palpation during a voluntary contraction [23]. Both electrodes (5 × 5 cm) were placed symmetrically to the sensor; the positive electrode (anode) was placed proximally, and the negative electrode (cathode) distally, 5 cm from the measuring point. Electrodes were self-adhesive (Compex Medical SA, Ecublens, Switzerland). The stimulation pulse was 1 ms, while the signal amplitude started at 30 mA. The electrical stimulation was applied with a TMG-S1 electrostimulator (Furlan Co. & Ltd., Ljubljana, Slovenia). For each pulse, the current amplitude was increased by 10 mA, until the maximal displacement of the muscle belly was reached [14]. To avoid fatigue or potentiation effects, a 15-s resting period was allowed between electrical stimuli. Of the total curves recorded for each lifeguard (ranged from 4–7), only the curve with the highest maximum radial displacement was included in the analysis. The same evaluator, who was experienced in taking these assessments, took all measurements. Maximal radial muscle-belly displacement (Dm) and contraction time between 10 and 90% Dm (Tc) were measured using TMG.

The participants’ blood lactate levels were measured before water rescue as basal level; lactate concentration was assessed again (1) after water rescue but previous to recovery protocol (pre-recovery lactate level) and (2) after the assigned recovery strategy (lactate post-recovery level). All measurements were made with LactateScout (SensLab GmbH, Leipzig, Germany) and expressed in mmol/l. Time elapsed between baseline lactate and lactate after water rescue were 10 min + personal time expended in water rescue (mean 3 min + 53 s). Time elapsed into lactate after water rescue, and lactate post-recovery was a time of TM (10 min) and recovery protocol (20 min).

Four separate RPE were administrated before water rescue, immediately after water rescue and finishing recovery interventions: an undifferentiated rating for the overall body (overall-RPE), a differentiated rating for peripheral perceptions of exertion in the legs (legs-RPE), a differentiated rating for peripheral perceptions of exertion in the arms (arms-RPE), and respiratory perceptions in the chest (ventilatory-RPE) [24]. The lifeguards had been familiarized with the instruments before the beginning of the study.

The raw data is presented as mean (M) with standard deviations (SD). Blood lactate levels were log-transformed as they were skewed. Log values of the blood lactate were approximately normally distributed and were therefore used in the inferential analyses. Paired sample *t*-tests were used to test for differences at baseline for lactate and RPE measures. A 2 × 2 RM-ANOVA (recovery protocol × time) was fitted for each of the variables. In case of significant main effects or interaction, post-hoc pairwise comparisons using Holm–Bonferroni correction for multiple comparisons were performed. The assumption of normality of residuals was not violated in any of the tests. R 3.6.1 was used for the analysis. Results were considered statistically significant when *p* < 0.05.

## 3. Results

Table 1 presents the baseline values of TMG previous to trial and the comparison between PR and ES recovery test in Lactate and RPE variables. The paired *t*-test revealed no significant difference between the two trials, with a small effect size for all variables. Follow-up equivalence testing showed that the effect size of the difference for all variables was significantly smaller than ±5 *d*.

Table 2 presents the pre- and post-recovery levels of lactate, RPEs, and TMG variables, together with the results of the repeated-measures analysis of variance for each variable. There was no change in TC or DM from pre to post recovery, and no difference between recovery protocols for either of the two variables.

For RPE overall, there were significant differences between the two recovery protocols, as well as a significant reduction in RPE score from pre to post recovery, but no significant interaction. Pairwise follow-up revealed no significant difference between protocols pre-recovery (t[31.2] = 1.21, *p* = 0.234, d = 0.28), significantly and moderately lower overall RPE scores for ES compared to PR (t[31.2] = 3.18, *p* = 0.016, d = 0.73), with large reductions of overall RPE between pre and post recovery, both for PR (t[25.3] = 8.40, *p* < 0.001, d = 1.93) and ES (t[25.3] = 9.89, *p* < 0.001, d = 2.27) protocols. 

For RPE legs and RPE arms, there were significant differences between the two recovery protocols, as well as a significant reduction in both RPE scores from pre to post recovery, but no significant interaction. Pairwise follow-up revealed no significant difference between protocols pre-recovery (t[35.3] = 0.64, *p* = 0.917, d = 0.15; t[35.9] = 1.51, *p* = 0.444, d = 0.35 for RPE legs and arms, respectively), and significantly and moderately lower both leg and arm RPE scores for ES compared to PR (t[35.3] = 3.35, *p* = 0.010, d = 0.77; t[35.9] = 2.58, *p* = 0.028, d = 0.59, respectively), with large reductions of legs and arms RPE between pre and post recovery, both for PR (t[31.2] = 6.84, *p* < 0.001, d = 1.57; t[35.1] = 5.29, *p* < 0.001, d = 1.21, respectively) and ES (t[31.2] = 9.12, *p* < 0.001, d = 2.09; t[35.1] = 6.30, *p* < 0.001, d = 1.45) protocols.

There was a significant reduction in ventilatory RPE score from pre to post recovery, but no significant difference between protocols or significant interaction. Pairwise follow-up revealed large reductions of ventilatory RPE between pre and post recovery, both for PR (t[31.4] = 3.56, *p* = 0.005, d = 0.82) and ES (t[31.4] = 5.22, *p* < 0.001, d = 1.20) protocols.

There was a significant reduction of blood lactate level after recovery, with significant difference in effect between the recovery protocols. Pairwise follow-up revealed no significant difference between protocols pre-recovery (t[34.8] = −0.886, *p* = 0.382, d = −0.20), significantly and moderately lower blood lactate for ES 4.77 ± 1.86 mmol·L^−1^ compared to PR protocol 6.27 ± 3.69 mmol·L^−1^ post-recovery (t[34.8] = 2.388, *p* = 0.045, d = 0.55), with large reductions of blood lactate between pre and post recovery, both for PR (t[30.1] = 5.007, *p* < 0.001, d = 1.15) and ES (t[30.1] = 7.716, *p* < 0.001, d = 1.77) protocols.

## 4. Discussion

The main finding of this study was that contrary to our first and second experimental hypotheses, ES did not alter neuromuscular fatigue (as indicated by TMG) or perceived effort (as indicated by RPE) compared to PR for lifeguards following a water rescue. However, in support of our third hypotheses, ES did reduce blood-lactate concentration. These findings indicate that ES might be effective for restoring acute metabolic fatigue after water rescue.

In the present study, two different TMG variables were used to evaluate the effect of ES and PR on contractile properties of the *rectus femoris* as this muscle plays an important role in the leg kick when swimming with fins [25]. Baseline and post rescue TMG data showed that rescue effort did not have an impact on contractile properties in lifeguards under meteorological conditions described. Both ES and PR maintained the contractile properties previous to the rescue. Results of present study are contrary to those observed by Mur Gimeno [20] and García-Manso et al. [21], who observed TGM variables restoration after cold-water immersion application in healthy men and soccer players, respectively. However, the present findings are in agreement with previous literature, as ES seems to be ineffective regarding neuromuscular parameters restoration, such as torque production capacity [26,27,28] or electromyography activity [26].

One of the expected principal effects of electrical stimulation is the reduction of the perceptions of pain and exertion ratings through stimulation of an analgesic effect that could aid toward the next event [8]. However, contrary to our hypothesis, the results of this study showed a large time effect on overall-RPE, ventilatory-RPE, legs-RPE, and arms-RPE from pre-rescue to post-recovery values both in PR group and ES group without significant differences between both recovery strategies. Present findings are challenging to place in perspective within the literature, as, to the best of our knowledge, no other study has investigated the effects of ES on perceived recovery after water rescue or swimming exercise. Nevertheless, these results are in partial disagreement with previous investigations using ES as a recovery tool in team-sports athletes [29,30,31]. Finberg et al. [30] observed significant benefits of ES in perceived recovery using the Total Quality Recovery Perceived Scale, compared with PR in team-sports athletes 24 h after a simulated team-game circuit. In addition, Taylor et al. [31] showed that the application of the ES resulted in reduced perceived muscle soreness at 24 h after a rugby training session. Discrepancies between cited studies and the present results may be explained by differences related to the study designs, activities to evoke fatigue, differences between samples, or differences in the time-line used for post-recovery evaluation (i.e., 24 h after fatiguing protocol vs. immediately after).

The lifeguards in this study reached a mean lactate level of over 10 mmol·L^−1^ after 200 m rescue with fins, which is in close agreement with the results of previous studies of water rescues of 150–200 m [4,5]. The results of this study showed a moderate recovery effect on lactate concentration after water rescue for the ES group compared with a PR group. These results are difficult to place in perspective with regard to the literature, because on the one hand no previous studies have used ES in lifesavers, and on the other hand, ES efficacy on blood lactate concentrations may be related to ES parameters used (pulse duration, current frequency, amplitude, and electrode placement) [9,32], target population [33], and type of fatiguing exercise or duration of recovery [32]. The present results are partially in agreement with a previous study in swimmers [8]. The effects of three post-exercise recovery interventions (sub-maximal swimming, ES, and PR) following 200-yards front crawl sprint were examined on blood lactate concentration. The results indicate that both active recovery strategies (sub-maximal swimming and ES) were the most efficient intervention to accelerate lactate removal. Thus, according to the results, 5 Hz and 0.25 milliseconds seem to be effective on lactate clearance when achieving a certain level of amplitude. Indeed, venous flow volume and velocity have been found to improve as amplitude is increased with low-frequency ES at a non-painful level [34]. According to present results, it appears that ES at 5 Hz and 0.25 milliseconds at painless motor amplitude could be an efficient strategy for lactate washout after water rescue. Thus, given the importance of lifeguards’ lactate concentration in rescue performance [5,35], the results of the present study have important practical implications and are useful for clarifying the most appropriate ES parameters for post-exercise short-term recovery.

This research presents limitations that must be taken into account to interpret the findings. The major limitation is that there are no additional variables directly related to lifesaving and functional outcome measures. In addition, it was not possible to carry out a test to assess the previous physical condition of the rescuers. Second, in the absence of scientific literature on muscle involvement in a water rescue with fins, the ES intervention on the legs muscles has been chosen as tow-in is performed only with lower limb propulsion and represents 2/3 of the time of lifesaving. Another limitation was the absence of sex-specific differences evaluation due to the low number of female participants in this study. Finally, lactate concentration was only measured at two time points post-exercise (pre-recovery and post-recovery protocol).

## 5. Conclusions

In summary, results of the present study indicate that the use of ES immediately after a water rescue ES may help in restoring blood lactate concentration. This recovery process could even take place from a watchtower, while the lifeguard watches the beach. Therefore, ES may constitute a simple and safe procedure, providing new perspectives in terms of intra-session recovery in lifesaving.

## Figures and Tables

**Table 1:** Description of tensiomyography (TMG) variables before the trial, and comparison before a water rescue of baseline levels of blood lactate and perceived exertion between recovery types.

TMG Variables *	**Data before Trial**								
	**M**					**Sd**			
TC	26.24					3.81			
DM	7.61					2.71			
Variables **	**PR**		**ES**		**Test of Difference 1**			**Equivalence Test 2**	
	**M**	**Sd**	**M**	**Sd**	**T**	***p*-Value**	**Cohen’s d**	***t***	***p*-Value**
Lactate	2.58	1.32	2.64	0.997	−0.115	0.909	−0.0258	2.12	0.024
RPE overall	2.05	1.50	1.77	1.88	0.370	0.715	0.0808	−1.92	0.035
RPE legs	2.23	1.85	2.33	2.31	−0.145	0.886	−0.0325	2.09	0.025
RPE arms	1.91	1.51	2.10	2.12	−0.493	0.628	−0.1102	1.74	0.049
RPE ventilatory	1.18	1.14	1.29	1.82	−0.260	0.798	−0.0581	1.98	0.031

DM = maximal radial muscle-belly displacement, TC = contraction time between 10 and 90% Dm. RPE = rate of perceived exertion, df = degrees of freedom. ^1^ Paired sample *t*-test. ^2^ Paired sample two one sided *t*-test (TOST). * Single measure before trial [passive recovery (PR) or electro recovery (ES) protoco], ** measure in baseline state before PR or ES test.

**Table 2:** Comparison after a water rescue, pre and post levels of blood lactate, and perceived exertion between recovery types.

	PR					ES					Anova					
Variables	Pre-Recovery Protocol		Post-Recovery Protocol			Pre-Recovery Protocol		Post-Recovery Protocol			Recovery		Time		Time × Recovery	
	M	Sd	M	Sd	% diff	M	Sd	M	Sd	% diff	*p*-Value	ES	*p*-Value	ES	*p*-Value	ES
Lactate	9.89	2.63	6.27	3.69	−36.6	10.9	3.06	4.77	1.86	−56.2	0.342	0.050	<0.001	0.757	0.020	0.267
RPE overall	7.59	1.71	3.57	2.40	−53.0	7.24	1.18	2.7	1.53	−62.7	0.017	0.278	<0.001	0.849	0.091	0.151
RPE legs	7.27	1.83	3.71	2.43	−49.0	7.05	1.72	2.65	1.66	−62.4	0.017	0.279	<0.001	0.836	0.053	0.192
RPE arms	6.18	1.68	3.29	1.79	−46.8	5.62	1.91	2.30	1.84	−59.1	0.011	0.306	<0.001	0.763	0.444	0.033
RPE ventilatory	5.64	2.40	3.00	2.76	−46.8	5.71	2.35	2.0	1.45	−65.0	0.081	0.160	<0.001	0.607	0.152	0.111
TC	27.5	3.31	27.8	3.76	1.1	27.3	2.97	28.1	4.82	2.9	0.943	0.000	0.441	0.033	0.537	0.021
DM	6.73	2.19	7.22	2.57	7.3	7.02	2.60	6.75	2.64	−4.0	0.740	0.006	0.672	0.010	0.087	0.154

PR = passive recovery, ES = electro recovery, RPE = rate of perceived exertion, TC = contraction time between 10 and 90% Dm, DM = maximal radial muscle-belly displacement.
